# The Type of Grain Counts: Effectiveness of Three Essential Oil-Based Nanoemulsions against *Sitophilus oryzae*

**DOI:** 10.3390/plants12040813

**Published:** 2023-02-11

**Authors:** Nickolas G. Kavallieratos, Giulia Bonacucina, Erifili P. Nika, Anna Skourti, Stavroula Kyriaki C. Georgakopoulou, Constantin S. Filintas, Anna Maria E. Panariti, Filippo Maggi, Riccardo Petrelli, Marta Ferrati, Eleonora Spinozzi, Diego Romano Perinelli, Angelo Canale, Giovanni Benelli

**Affiliations:** 1 Laboratory of Agricultural Zoology and Entomology, Department of Crop Science, Agricultural University of Athens, 75 Iera Odos Street, 11855 Athens, Greece; 2 Chemistry Interdisciplinary Project (ChIP) Research Center, School of Pharmacy, University of Camerino, Via Madonna delle Carceri 9/B, 62032 Camerino, Italy; 3 Faculty of Biology, Institute of Zoology, University of Belgrade, Studenstki trg 16, 11000 Belgrade, Serbia; 4 Department of Agriculture, Food and Environment, University of Pisa, Via del Borghetto 80, 56124 Pisa, Italy

**Keywords:** barley, grain protectant, green insecticide, maize, nanopesticide, oats, stored-product pest control

## Abstract

Essential oil (EO)-based nanoemulsions (NEs) are promising grain protectants in the management of stored-product pests. However, the potential impact of the stored-grain species on the green insecticide effectiveness has been poorly studied. In this study, two concentrations of EO-based NEs from *Carlina acaulis* L., *Mentha longifolia* (L.) Huds., and *Hazomalania voyronii* (Jum.) Capuron were evaluated as insecticides against the major stored-product pest *Sitophilus oryzae* (L.) on barley, oats, and maize kernels. The *C. acaulis* EO-based NE applied at 1000 ppm on barley achieved the highest mortality, killing 94.4% of *S. oryzae* adults after a 7-day exposure, followed by 1000 ppm of *H. voyronii* EO-based NE (83.3%). The lowest mortality (1.1%) was recorded with 500 ppm of *M. longifolia* EO-based NE on maize after the same interval. All tested NEs exhibited elevated efficacy when applied on barley, while mortalities were lower on oats and maize. Furthermore, *C. acaulis* EO-based NE was the most effective when applied on all commodities, followed by *H. voyronii* and *M. longifolia* EO-based NEs. Overall, our results highlighted the significant impact of the stored cereal on the insecticidal effectiveness of EO-based NE used for stored-product pest control. *Sitophilus oryzae* adults on barley can be adequately controlled through the application of *C. acaulis* and *H. voyronii* EO-based NEs.

## 1. Introduction

Essential oils (EOs) are complex mixtures of volatile, lipophilic substances, often characterized by a strong odor, and showing interesting bioactivities against viruses, bacteria, fungi, and arthropod pests and vectors, among others [1,2,3,4]. Therefore, their application on stored-product pest management constitutes a potential alternative to the overuse of synthetic insecticides [3,4,5]. To enhance the properties of plant EOs, they can be encapsulated into nanoemulsions (NEs), i.e., dispersions of oil nanodroplets in water that are kinetically stable thanks to the presence of surfactants (surfactant-to-oil ratio generally between 1 and 2) [6]. NEs are more easily dispersible in water than pure EOs; therefore, they can interact with greater ease than the EOs with the target sites [6,7].

*Sitophilus oryzae* (L.) is a primary pest globally, attacking many types of stored grains, as well as pulses and nuts [8,9]. It is a small destructive beetle species (2.5–4 mm), causing great economic damages worldwide, especially in warm climate regions [8,10,11]. Despite *S. oryzae* being a key insect in storages, the currently used synthetic insecticides face resistance and tolerance issues concerning its effective management [4,12,13,14].

Concerning the pesticidal potential of EOs against *S. oryzae*, many plant species have been tested previously mainly as contact insecticides on filter paper or as fumigants [15,16,17,18,19,20,21]. For example, *Mentha spicata* L., *Mentha rotundifolia* (L.) Huds., and *Mentha longifolia* (L.) Huds. EOs have been tested on filter paper, killing 95% of *S. oryzae* after 18 h exposure [17]. Furthermore, *Melaleuca rhaphiophylla* Schauer EO was more effective against *S. oryzae* as a fumigant than by contact [21]. Of note, only a few studies focus on the utilization of EOs as grain protectants against *S. oryzae* adults. For instance, 100% mortality of *S. oryzae* adults was recorded after exposing them for 6 days to *Carlina acaulis* L. EO-treated stored wheat [4]. When EOs from *Pinus roxburghii* Sarg., *Psidium guajava* L., and *Haplophyllum tuberculatum* (Forsk.) Ad. Juss. were applied on wheat, they killed all exposed beetles after 14 days of exposure [22]. Studies including EO-based NEs for the management of *S. oryzae* adults are even more scarce. Adak et al. [23] documented that the eucalyptus EO provided lower mortality rates if compared to its NE applied on concrete. Choupanian et al. [24] reported that the NE of *Azadirachta indica* A. Juss killed more *S. oryzae* adults than the EO when applied on filter paper. Recently, Choupanian and Omar [25] pointed out the insecticidal efficacy of *A. indica* EO-based NE on wheat and rice.

However, despite these efforts, little is known about potential variations in the EO-based NE insecticidal efficacy when distributed on different stored cereals. To tackle this research challenge, herein we selected three EOs with proven insecticidal activity, formulating them in NEs, and assessing their effectiveness against *S. oryzae* when distributed on barley, oats, and maize grains. *Carlina acaulis* is a perennial species typically found on warm and calcareous mountainous areas of Central and Southern Europe [26]. It is a thoroughly documented medicinal herb and has been used in traditional medicine for its diuretic, cholagogic, anti-inflammatory, antibiotic, and laxative functions [27,28]. It is a quite common ingredient for culinary uses across its distributional areas [27,29]. Parts of this plant can be consumed raw, cooked, or as decoction [27,29]. Its root EO has been reported as highly effective against important groups of pests, including beetles, houseflies, moths, and mosquitoes [4,30,31,32,33,34,35]. *Mentha longifolia* is a widespread wild perennial herb, native to temperate regions across Eurasia and Africa [36,37]. It is a pungent scented plant, with erect stems and spiked multi-flowered inflorescences [38], which is extensively consumed for its medicinal properties, as well as a spice and an infusion in culinary practices [39,40,41,42]. Its EO has antioxidant, anti-inflammatory, antispasmodic, analgesic, and anticancer activities from a human healthcare point of view [43]. Earlier research has documented its antiparasitic, antimicrobial, and insecticidal properties [44,45]. For example, *M. longifolia* EO has elevated insecticidal activities against *Tribolium castaneum* (Herbst) and *Callosobruchus maculatus* (F.), when applied on filter paper [44]. *Hazomalania voyronii* (Jum.) Capuron is an endemic plant species of Western Madagascar located in dry forests [46]. The bark of this plant has been used in local Malagasy folk medicine to treat wounds and infections [47]. A bark was being used in conjunction with chloroquine by local people in western Madagascar to treat malaria, since the extract of *H. voyronii* has potent anti-plasmodial properties [48]. Moreover, the major compound of the species, i.e., perilla aldehyde, is used in food industry [49]. Its EOs and NEs are effective against insect vectors, agricultural pests, and stored product beetles [46,49].

Although the EO-based NEs of *M. longifolia, C. acaulis,* and *H. voyronii* have been assessed on grains for managing pests attacking stored products [49,50,51], there are no data available for their efficacy when applied on various types of cereals against *S. oryzae*. Therefore, the objective of this research was to evaluate the insecticidal effectiveness of *M. longifolia, C. acaulis,* and *H. voyronii* EO-based NEs against adults of *S. oryzae* when formulated on barley, oats, and maize kernels.

## 2. Results

*Carlina acaulis, M. longifolia,* and *H. voyronii* EOs yields, as well as their chemical compositions, were in line with those of Kavallieratos et al. [52]. Specifically, the polyacetylene carlina oxide (94.6%) was the main compound of the *C. acaulis* EO, followed by a minor percentage of benzaldehyde (3.1%). *Mentha longifolia* EO was mainly composed of piperitenone oxide (61.1%), and minor percentages of myrcene (10.8%), 1,8-cineole (5.3%), and limonene (5.3%). Perilla aldehyde (43.0%) was the dominant compound of *H. voyronii* EO, followed by 1,8-cineole (33.2%) and limonene (13.0%).

The three tested NEs were prepared according to the procedure and the quantitative composition of the formulation previously optimized in terms of encapsulated EO and polysorbate 80 (as emulsifier) ratios [49,50,51]. Particle size, expressed as the mean hydrodynamic diameter (Z-average), and particle size distribution, expressed as polydispersity index (PDI) of the disperse oil droplets, were 53.51 ± 1.10 nm, 0.382 ± 0.004 for *H. voyronii* EO-based NE; 102.26 ± 12.4 nm, 0.342 ± 0.036 for *C. acaulis* EO-based NE; and 142.46 ± 1.11 nm, 0.269 ± 0.012 for *M. longifolia* EO-based NE. Values are in accordance with those previously reported [52].

Concerning insecticidal assays, exposures (between, within), all main effects and the corresponding interactions were significant (Table 1). When the insecticides were applied on barley, 1000 ppm of *C. acaulis* and *H. voyronii* EO-based NEs, as well as pirimiphos-methyl killed 61.1, 44.4, and 53.3% of the exposed individuals, 5 days post-exposure, respectively (Table 2). At the termination of the bioassays, 1000 ppm of *C. acaulis* EO-based NE led to the highest value of mortality (94.4%), followed by pirimiphos-methyl (88.9%) and 1000 ppm of *H. voyronii* EO-based NE (83.3%). The lowest mortality was noticed testing 500 ppm of *M. longifolia* EO-based NE, which did not exceed 22.2% at the end of the experimentation. The remaining insecticides, i.e., 500 ppm of *C. acaulis* and *H. voyronii* EO-based NEs and 1000 ppm of *M. longifolia* EO-based NE, killed 35.6–46.7% in the same exposure.

The tested insecticides applied on oats, provided lower mortality rates in comparison to those distributed on barley (Table 3). On the fourth day of the trials, pirimiphos-methyl caused death to 30.0% of *S. oryzae* while all the other tested insecticides killed 1.1–6.7% of the individuals. Pirimiphos-methyl caused 76.7% mortality, followed by 1000 ppm *C. acaulis* EO-based NE (43.3%) and 1000 ppm *H. voyronii* EO-based NE (27.8%), after 7 days of exposure. The other tested insecticides caused the death of 11.1 (500 ppm *H. voyronii* EO-based NE)-20.0% (1000 ppm *M. longifolia* EO-based NE) of *S. oryzae* adults, at the termination of the trials.

Concerning maize, the tested insecticides exhibited the lowest efficacy among the other tested grain commodities (Table 4). The highest mortality was recorded after the exposure for 7 days at pirimiphos-methyl treated maize, reaching 57.8%. Only 1000 ppm *C. acaulis* EO-based NE were able to kill 17.8% of the exposed *S. oryzae*, while the remaining five insecticides did not exceed 7.8% mortality 7 days post-exposure. The lowest mortality was observed for *M. longifolia* EO-based NE, not exceeding 1.1 and 3.3% at the termination of the bioassays, for 500 ppm and 1000 ppm, respectively.

## 3. Discussion

Our results pointed out the high efficacy of *C. acaulis* EO-based NE distributed at 1000 ppm on barley against *S. oryzae* adults. Currently, this pest has developed resistance to several synthetic insecticides used as grain protectants, fumigants, or contact insecticides, i.e., chlorpyrifos-methyl, cypermethrin, spinosad, permethrin, phosphine, malathion, and pirimiphos-methyl [53,54,55]. For instance, a geographical strain of *S. oryzae* from Rostov was 2.5-fold more phosphine resistant than the laboratory strain to achieve 99.9% mortality [54]. Furthermore, earlier research has indicated that this species is hard to manage using alternative, eco-friendly insecticides. For example, plant powders from nine plant species, applied on wheat, provided 0.0–40.0% mortality after a 7-day exposure [56]. Furthermore, bay leaf, custard apple, neem, and tobacco leaf powders, as well as citronella EOs applied on wheat, maize, and paddy rice, killed 0.0–25.0% of *S. oryzae*, 7 days post-exposure [57]. Five plant extracts applied on wheat exhibited low to moderate mortalities, ranging from 20.00 to 56.66% after 7 days of exposure [58]. In this scenario, the insecticidal activity caused by 1000 ppm of *C. acaulis* EO-based NE (94.4%) and 1000 ppm of *H. voyronii* EO-based NE (83.3%) represent an important outcome in the framework of finding a sustainable insecticidal tool for managing this species.

The EO-based NEs tested here have been used against several stored-product pest species as wheat protectants, leading to significant mortalities. As a general trend, it should be noted that all these insecticides exhibited variable efficacy depending on their concentration as well as the target species/developmental stage [49,50,51]. For example, 500 ppm of *M. longifolia* EO-based NE killed 24.4% of *Acarus siro* L. adults, a significantly lower killing percentage than 1000 ppm which caused the death to 82.2% individuals, 7 days post-exposure [51]. Furthermore, 1000 ppm of *H. voyronii* EO-based NE killed 100% of *Tenebrio molitor* L. adults but only 10.3% of the larvae [49]. *Carlina acaulis* and *M. longifolia* EO-based NEs did not provide total (100%) mortality on *T. molitor* adults, but 85.2 and 91.1%, respectively [50,51]. The differences in the NEs’ efficacy could be linked to the different EOs encapsulated and, consequently, to their diverse modes of action. Firstly, *C. acaulis* EO, which already demonstrated its great potential on a wide spectrum of stored-product pests [35], was mainly characterized by the presence of carlina oxide (94.6% of the total composition). The mode of action of this polyacetylene is still obscure. However, this class of compounds is characterized by high instability and reactivity, linked to the presence of C-C triple bonds. In detail, these molecules are particularly sensitive to variations of pH and are subjected to fast oxidation when exposed to UV light [59]. Regarding this feature, these compounds are considered photosensitizers since they are activated by sunlight wavelengths smaller than 400 nm with a consequent enhancement of their toxicity also achieved by photodynamic disruption of membranes [60,61]. This mechanism of action has been proposed also for carlina oxide, whose C-C triple bond seems to lead to the formation of radicals after UV-light exposure [62]. Furthermore, *M. longifolia* EO was characterized by the presence of piperitenone oxide and myrcene (61.1 and 10.8% respectively). Piperitenone oxide, which is a monoterpenoid bearing an epoxide group, is a characterizing component of different *Mentha* L. EOs and already displayed a strong action on stored-product pests [51,63], but also on mosquitoes as *Anopheles stephensi* Liston, on which it had reproduction retardant, repellent, and toxic effects [64]. The biological activities of piperitenone oxide have been linked to the epoxide moiety, which is responsible for the interaction with neurotransmitters, proteins, and nucleic acids [65]. In addition, myrcene has also been reported as active against some stored-products pests [66], and its mode of action seems to be linked to a neurotoxic effect [67]. Lastly, *H. voyronii* EO was mainly constituted by limonene (13.0%), 1,8-cineole (33.2%), and perilla aldehyde (43.0%). Perilla aldehyde showed contact and fumigant toxicities [68], which are linked to its acetylcholinesterase (AChE) inhibitory activity, already reported also on *S. oryzae* [69]. The action of perilla aldehyde could be enhanced by 1,8-cineole, whose toxic action on stored-product pests has already been reported as probably linked to the AChE inhibition [70,71]. Regarding limonene, this compound seems to act through a neurotoxic action [67].

Interestingly, 1000 ppm of *C. acaulis* EO killed all *S. oryzae* in a 6-days period [4]. Given that the *C. acaulis* EO-based NE used in the current study contained 6% (*w*/*w*) *C. acaulis* EO, the actual concentration of the EO contained into the NE was ~17 times lower than into the *C. acaulis* EO. This is one other important finding since such a small amount of EO could almost suppress *S. oryzae* adults on barley. Concerning *M. longifolia*, when the EO was applied on wheat, it killed more *T. molitor*, *Tribolium castaneum* (Herbst), *Oryzaephilus surinamensis* (L.), *Tribolium confusum* Jacquelin du Val, and *A. siro* individuals than its EO-based NE, but it should be noted that the EO concentration in the NE was 10 times lower [51].

The grain species plays a key role when it is directly treated with insecticides, but to the best of our knowledge, no records are available about EO-NE. Earlier research has documented this phenomenon for different insecticides and stored-product pests. For example, Kavallieratos et al. [72] tested spinosad, deltamethrin, silicoSec, and pirimiphos-methyl, against *T. molitor* adults and larvae on maize, barley, and wheat. All tested insecticides provided higher mortalities on barley, followed by wheat and maize. Similarly, when spinosad, pirimiphos-methyl, cypermethrin, silicoSec, and deltamethrin were applied on rough rice, maize, wheat, and barley against *Trogoderma granarium* Everts adults and larvae [73], they provided variable mortalities. Arthur [74] found that methoprene treated on brown and rough rice could significantly reduce the number of *Sitotroga cerealella* (Olivier) progeny, contrary to methoprene treated on maize. Treated wheat killed more individuals, followed by barley, maize, and rough rice, for both developmental stages. Concerning *S. oryzae*, etofenprox treated on barley provided the highest mortality in comparison with maize, wheat, whole rice, and oats [75]. The grain texture may be responsible for this variation of results. Stored-product insects, such as *Rhyzopertha dominica* (F.), face difficulties walking on smooth surfaces [76]. The fact that maize is considerably smoother than other types of grains [77] could partially explain the lower mortalities of *S. oryzae* on maize vs. barley or oats, since insects may not walk so easily, reducing the probability of their contact with the insecticide. Furthermore, grains may exhibit different insecticide-adherence capability [78]. For example, triticale, maize, peeled barley, whole barley, rye, wheat, rice, and oats could adhere variably diatomaceous earths (DEs) [79]. Maize had the lowest adherence ability (<10%), while whole barley and oat had relatively high adherence ability (~85% and ~78%, respectively), and subsequently resulted in higher mortality rates if compared to the maize [80]. Whether the efficacy of the tested EO-based NEs is linked with their adherence and/or the level of ability of insects to walk on kernels merits further investigation.

## 4. Materials and Methods

### 4.1. Encapsulation of Essential Oils into Nanoemulsions

*Carlina acaulis, M. longifolia,* and *H. voyronii* EOs employed in the formulations were obtained and characterized as in the previous study of Kavallieratos et al. [52]. They were encapsulated into NEs using high-pressure homogenization method (French pressure cell press; American Instrument Company, Silver Spring, MY, USA). Their compositions were: (i) 6% (*w*/*w*) *C. acaulis*, 4% (*w*/*w*) polysorbate 80, 90% (*w*/*w*) water; (ii) 10% (*w*/*w*) *M. longifolia*, 2% (*w*/*w*) ethyl oleate, 3% (*w*/*w*) polysorbate 80, 85% (*w*/*w*) water; (iii) 6% (*w*/*w*) *H. voyronii*, 4% (*w*/*w*) polysorbate 80, 90% (*w*/*w*) water. The NEs were prepared and characterized as reported in the literature [49,50,51].

### 4.2. Insect Species

A Greek strain of *S. oryzae* was cultured on whole wheat grains at 30 °C and 65% relative humidity (RH), without the presence of a light source [80,81]. Unsexed *S. oryzae* adults, not older than two weeks, were tested [80,81].

### 4.3. Grains

Maize, *Zea mays* L. (var. Dias), barley, *Hordeum vulgare* L. (var. Persephone), and oats, *Avena sativa* L. (var. Cassandra), kernels were free from impurities, infestations and pesticides. Their moisture content was adjusted at 13.5% (by heating or adding distilled water) via a calibrated moisture meter (mini GAC plus, Dickey-John Europe S.A.S., Colombes, France), prior to trials [72].

### 4.4. Bioassays

The tested concentrations of *M. longifolia* (10% *w*/*w*), *C. acaulis* (6% *w*/*w*), and *H. voyronii* EO-based NEs (6% *w*/*w*) were selected prior the experiments at 500 μL/kg grains (=500 ppm) and 1000 μL/kg grains (=1000 ppm), through preliminary tests. A total 750 μL volume of insecticides was formed by mixing 125 μL of NE with 625 μL of water (for 500 ppm) and 250 μL of NE with 500 μL of water (for 1000 ppm). To conduct the spraying, thin layers of barley, oats, or maize (0.25 kg), were laid on disks, and, subsequently, each of the insecticides were applied with a unique BD-134K airbrush (Fengda, UK). Concerning controls, extra grain lots treated with (i) water, (ii) carrier control 1 (4% *w*/*w* surfactant dispersed in water), (iii) carrier control 2 (water 97% and polysorbate 80 3%), (iv) carrier control 3 (water 95%, ethyl oleate 2%, and polysorbate 80 3%), and (v) positive control i.e., pirimiphos-methyl, at the label dose of 5 μL/kg grains (=5 ppm) (label dose) (Actellic EC, containing 50% active ingredient (a.i.), Syngenta, Anthousa, Greece), were treated on different grain lots, on separate disks with unique BD-134K airbrushes for each control formulation. Afterwards, treated grain lots were transferred separately on 1-L containers made of glass for 10 min of handshaking to disperse the insecticides/controls further evenly onto the entire grain mass. Filter papers were utilized to weigh three samples (10 g each) with a compact balance (Precisa XB3200D, Alpha Analytical Instruments, Gerakas, Greece). The samples were conveyed into glass vials (7.5 cm diameter × 12.5 cm height), with hole-bearing caps (1.5 cm diameter). The holes were cloth-covered and enabled the aeration of their spaces. The top internal sides of the vials were polished by polytetrafluoroethylene (Sigma-Aldrich Chemie GmbH, Taufkirchen, Germany) to stop beetles leaving the vials. Thereupon, 10 beetles were transferred into the vials containing the grains. Incubators set at 30 °C and 65% RH held the prepared vials for the whole period of the experiment. Data of mortality were noted daily after 4, 8, and 16 h for a period of a week. Inspection was conducted under a stereomicroscope (Olympus SZX9, Bacacos S.A., Athens, Greece) with a brush that lightly nudged the insect individuals. Upon no movement, the beetle was declared dead. Each insecticide and control had its unique brush to avoid cross contamination. All above-reported procedures were repeated again twice using new grains, vials, and insect individuals. The total number of the participated individuals was 2.970 (10 individuals × 3 replicates × 3 subreplicates × 3 types of grain × 11 insecticides/controls).

### 4.5. Data Analysis

Negative controls (water and carrier controls) provided <5% mortalities; therefore, no corrections were applied to mortality data. For variance normalization, data were log (x + 1) transformed before analysis [82,83]. Repeated measures model was used in the analysis [84]. Mortality corresponded to response variable. Grain and insecticide corresponded to main effects. Associated interactions of grain and tested insecticide were included into the analysis. The JMP 16.2 software was utilized to conduct the entire analysis [85]. Means were separated at 0.05 level of significance by the Tukey–Kramer honestly significant difference test [86].

## 5. Conclusions

In conclusion, this work firstly shed light on the insecticidal efficacy of EO-based NEs when distributed on different stored grains. The *C. acaulis* EO-based NE was highly effective against *S. oryzae* adults, but only when applied on barley. Its efficacy was correlated to the presence of the polyacetylene carlina oxide. This is a promising insecticide owing to its likely capacity to give rise to radical species producing damages in the insect tissues, especially under the UV light. *Hazomalania voyronii* EO-based NE distributed on barley could adequately control *S. oryzae*, while mortality on oats and maize was low to moderate. Its insecticidal effects are linked with the presence of perilla aldehyde, 1,8-cineole, and limonene, that, taken together, might produce neurotoxic effects. In general, *M. longifolia* EO-based NE provided lower mortality rates when distributed on all types of grains, if compared to the other tested EO-based NEs. Although this EO was characterized by the neurotoxic piperitenone oxide, it is likely that its activity could be weakened by the presence of other harmless components. The NE effectiveness rate from the most to the least effective insecticides was *C. acaulis* > *H. voyronii* > *M. longifolia*. The three types of grains tested here are classified from an effectiveness point of view against *S. oryzae* as follows: barley > oats > maize (from the highest to the lowest documented mortalities). Although EO-based NEs represent an effective pest management tool against some noxious arthropods, further research is still needed to understand how to enhance their pesticidal properties on a wide spectrum of stored foodstuffs, to unravel their full potential against key stored-product pests. It should be noted that the application of NE on grains leaves no residues on the final product due to standard milling procedure of the raw commodities [87]. Last, but not least, the toxicological profile of natural substances needs additional research efforts. Interestingly, *C. acaulis* EO is safe to non-target mammals [88].

## Figures and Tables

**Table 1:** MANOVA parameters for the main effects and associated interactions leading to the observed mortality rates of *Sitophilus oryzae* adults between and within exposure intervals (error DF = 168).

Between Exposure Intervals	DF	*F*	*p*
Intercept	1	1499.5	<0.01
Insecticide	6	67.5	<0.01
Grain	2	149.3	<0.01
Insecticide x grain	12	5.6	<0.01
Within exposure intervals			
Exposure	9	303.0	<0.01
Exposure x insecticide	54	7.0	<0.01
Exposure x grain	18	20.1	<0.01
Exposure x insecticide x grain	108	2.5	<0.01

**Table 2:** Mean (%) mortality ± standard error (SE) of *Sitophilus oryzae* adults after 4 h, 8 h, 16 h, 1 day, 2 days, 3 days, 4 days, 5 days, 6 days, and 7 days in barley treated with 6% (*w*/*w*) *Carlina acaulis*, 10% (*w*/*w*) *Mentha longifolia,* and 6% (*w*/*w*) *Hazomalania voyronii* essential oil-based nanoemulsions at two different concentrations. Pirimiphos-methyl was the positive control.

Tested Product	4 h	8 h	16 h	1 Day	2 Days	3 Days	4 Days	5 Days	6 Days	7 Days	*F*	*p*
*C. acaulis* NE 500 ppm	0.0 ± 0.0 D	0.0 ± 0.0 D	0.0 ± 0.0 D	0.0 ± 0.0 Db	0.0 ± 0.0 Dc	5.6 ± 1.8 Cb	8.9 ± 2.0 Cbc	16.7 ± 1.7 Bb	28.9 ± 2.0 ABbc	45.6 ± 1.8 Abc	72.9	<0.01
*C. acaulis* NE 1000 ppm	0.0 ± 0.0 E	0.0 ± 0.0 E	0.0 ± 0.0 E	0.0 ± 0.0 Eb	6.7 ± 1.7 Dab	23.3 ± 2.9 Ca	33.3 ± 4.1 BCa	61.1 ± 4.6 ABa	80.0 ± 2.9 Aa	94.4 ± 1.8 Aa	206.5	<0.01
*M. longifolia* NE 500 ppm	0.0 ± 0.0 B	0.0 ± 0.0 B	0.0 ± 0.0 B	0.0 ± 0.0 Bb	0.0 ± 0.0 Bc	0.0 ± 0.0 Bc	2.2 ± 1.5 Bd	4.4 ± 1.8 Bc	15.6 ± 3.4 Ad	22.2 ± 5.2 Ad	17.1	<0.01
*M. longifolia* NE 1000 ppm	0.0 ± 0.0 C	0.0 ± 0.0 C	0.0 ± 0.0 C	0.0 ± 0.0 Cb	0.0 ± 0.0 Cc	0.0 ± 0.0 Cc	2.2 ± 1.5 Cd	12.2 ± 1.5 Bb	22.2 ± 4.9 ABcd	35.6 ± 4.4 Ac	110.7	<0.01
*H. voyronii* NE 500 ppm	0.0 ± 0.0 D	0.0 ± 0.0 D	0.0 ± 0.0 D	0.0 ± 0.0 Db	0.0 ± 0.0 Dc	0.0 ± 0.0 Dc	8.9 ± 3.1 Ccd	20.0 ± 3.3 Bb	25.6 ± 3.8 ABbcd	46.7 ± 5.0 Abc	80.6	<0.01
*H. voyronii* NE 1000 ppm	0.0 ± 0.0 E	0.0 ± 0.0 E	0.0 ± 0.0 E	3.3 ± 1.7 DEab	5.6 ± 1.8 Db	20.0 ± 2.9 Ca	27.8 ± 4.7 BCab	44.4 ± 3.4 ABCa	56.7 ± 3.3 ABab	83.3 ± 3.3 Aab	81.1	<0.01
Pirimiphos-methyl	0.0 ± 0.0 F	0.0 ± 0.0 F	0.0 ± 0.0 F	4.4 ± 1.8 Ea	14.4 ± 2.9 Da	25.6 ±3.4 CDa	34.4 ± 3.8 BCa	53.3 ± 5.0 ABCa	72.2 ± 6.0 ABa	88.9 ± 3.9 Aab	100.0	<0.01
*F*	-	-	-	4.4	16.2	69.8	17.6	32.7	13.6	15.7		
*p*	-	-	-	<0.01	<0.01	<0.01	<0.01	<0.01	<0.01	<0.01		

Within each row, means followed by the same uppercase letter are not significantly different (DF = 9, 89; Tukey’s HSD test at *p* = 0.05). Within each column, means followed by the same lowercase letter are not significantly different (DF = 6, 62; Tukey’s HSD test at *p* = 0.05). Where dashes exist, no analysis was conducted.

**Table 3:** Mean (%) mortality ± standard error (SE) of *Sitophilus oryzae* adults after 4 h, 8 h, 16 h, 1 day, 2 days, 3 days, 4 days, 5 days, 6 days, and 7 days in oats treated with 6% (*w*/*w*) *Carlina acaulis*, 10% (*w*/*w*) *Mentha longifolia,* and 6% (*w*/*w*) *Hazomalania voyronii* essential oil-based nanoemulsions at two different concentrations. Pirimiphos-methyl was the positive control.

Tested Product	4 h	8 h	16 h	1 Day	2 Days	3 Days	4 Days	5 Days	6 Days	7 Days	*F*	*p*
*C. acaulis* NE 500 ppm	0.0 ± 0.0 B	0.0 ± 0.0 B	0.0 ± 0.0 B	0.0 ± 0.0 B	0.0 ± 0.0 Bb	0.0 ± 0.0 Bb	2.2 ± 1.5 Bb	3.3 ± 1.7 Bb	13.3 ± 2.4 Ab	18.9 ± 2.0 Abc	41.1	<0.01
*C. acaulis* NE 1000 ppm	0.0 ± 0.0 B	0.0 ± 0.0 B	0.0 ± 0.0 B	1.1 ± 1.1 B	1.1 ± 1.1 Bb	3.3 ± 1.7 Bb	3.3 ± 1.7 Bb	3.3 ± 1.7 Bb	21.1 ± 2.6 Aab	43.3 ± 5.8 Aab	26.7	<0.01
*M. longifolia* NE 500 ppm	0.0 ± 0.0 B	0.0 ± 0.0 B	0.0 ± 0.0 B	0.0 ± 0.0 B	0.0 ± 0.0 Bb	0.0 ± 0.0 Bb	1.1 ± 1.1 Bb	3.3 ± 2.4 Bb	11.1 ± 3.1 Abc	14.4 ± 3.4 Ac	16.6	<0.01
*M. longifolia* NE 1000 ppm	0.0 ± 0.0 C	0.0 ± 0.0 C	1.1 ± 1.1 C	2.2 ± 2.2 C	3.3 ± 2.4 BCb	3.3 ± 2.4 BCb	6.7 ± 2.9 ABCb	10.0 ± 3.7 ABCb	16.7 ± 5.0 ABbc	20.0 ± 5.5 Ac	6.5	<0.01
*H. voyronii* NE 500 ppm	0.0 ± 0.0 C	0.0 ± 0.0 C	0.0 ± 0.0 C	0.0 ± 0.0 C	0.0 ± 0.0 Cb	0.0 ± 0.0 Cb	1.1 ± 1.1 BCb	2.2 ± 2.2 BCb	5.6 ± 2.4 Bc	11.1 ± 2.0 Ac	11.6	<0.01
*H. voyronii* NE 1000 ppm	0.0 ± 0.0 E	0.0 ± 0.0 E	0.0 ± 0.0 E	0.0 ± 0.0 E	0.0 ± 0.0 Eb	2.2 ± 1.5 DEb	5.6 ± 1.8 CDb	7.8 ± 2.2 BCb	16.7 ± 2.4 ABb	27.8 ± 3.6 Abc	25.0	<0.01
Pirimiphos-methyl	0.0 ± 0.0 E	0.0 ± 0.0 E	0.0 ± 0.0 E	4.4 ± 2.4 DE	10.0 ± 2.9 CDa	22.2 ± 4.0 BCa	30.0 ± 4.4 ABa	50.0 ± 4.1 ABa	62.2 ± 5.2 Aa	76.7 ± 3.7 Aa	54.9	<0.01
*F*	-	-	1.0	2.0	7.0	12.1	9.3	9.9	7.8	9.8		
*p*	-	-	0.44	0.09	<0.01	<0.01	<0.01	<0.01	<0.01	<0.01		

Within each row, means followed by the same uppercase letter are not significantly different (DF = 9, 89; Tukey’s HSD test at *p* = 0.05). Within each column, means followed by the same lowercase letter are not significantly different (DF = 6, 62; Tukey’s HSD test at *p* = 0.05). Where no letters exist, no significant differences were recorded. Where dashes exist, no analysis was conducted.

**Table 4:** Mean (%) mortality ± standard error (SE) of *Sitophilus oryzae* adults after 4 h, 8 h, 16 h, 1 day, 2 days, 3 days, 4 days, 5 days, 6 days, and 7 days in maize treated with 6% (*w*/*w*) *Carlina acaulis*, 10% (*w*/*w*) *Mentha longifolia,* and 6% (*w*/*w*) *Hazomalania voyronii* essential oil-based nanoemulsions at two different concentrations. Pirimiphos-methyl was the positive control.

Tested Product	4 h	8 h	16 h	1 Day	2 Days	3 Days	4 Days	5 Days	6 Days	7 Days	*F*	*p*
*C. acaulis* NE 500 ppm	0.0 ± 0.0 C	0.0 ± 0.0 C	0.0 ± 0.0 C	0.0 ± 0.0C	0.0 ± 0.0 Cb	0.0 ± 0.0 Cb	0.0 ± 0.0 Cb	2.2 ± 1.5 BCb	5.6 ± 1.8 ABbc	7.8 ± 1.5 Abc	10.8	<0.01
*C. acaulis* NE 1000 ppm	0.0 ± 0.0 B	0.0 ± 0.0B	0.0 ± 0.0 B	0.0 ± 0.0 B	0.0 ± 0.0 Bb	0.0 ± 0.0 Bb	2.2 ± 1.5 Bb	2.2 ± 1.5 Bb	8.9 ± 2.6 Ab	17.8 ± 4.0 Aab	14.4	<0.01
*M. longifolia* NE 500 ppm	0.0 ± 0.0	0.0 ± 0.0	0.0 ± 0.0	0.0 ± 0.0	0.0 ± 0.0 b	0.0 ± 0.0 b	0.0 ± 0.0 b	0.0 ± 0.0b	0.0 ± 0.0c	1.1 ± 1.1d	1.0	0.45
*M. longifolia* NE 1000 ppm	0.0 ± 0.0	0.0 ± 0.0	0.0 ± 0.0	0.0 ± 0.0	0.0 ± 0.0 b	0.0 ± 0.0b	0.0 ± 0.0 b	0.0 ± 0.0 b	1.1 ± 1.1 c	3.3 ± 2.4 cd	1.7	0.10
*H. voyronii* NE 500 ppm	0.0 ± 0.0 B	0.0 ± 0.0 B	0.0 ± 0.0 B	0.0 ± 0.0 B	0.0 ± 0.0 Bb	0.0 ± 0.0 Bb	0.0 ± 0.0 Bb	0.0 ± 0.0 Bb	2.2 ± 1.5 ABbc	4.4 ± 2.4 Acd	3.0	<0.01
*H. voyronii* NE 1000 ppm	0.0 ± 0.0 B	0.0 ± 0.0 B	0.0 ± 0.0 B	0.0 ± 0.0 B	0.0 ± 0.0 Bb	0.0 ± 0.0 Bb	2.2 ± 1.5 ABb	3.3 ± 2.4 ABb	5.6 ± 2.4 ABbc	6.7 ± 2.4 Abcd	4.1	<0.01
Pirimiphos-methyl	0.0 ± 0.0 D	0.0 ± 0.0 D	0.0 ± 0.0 D	0.0 ± 0.0 D	7.8 ± 2.8 Ca	11.1 ± 3.5 Ca	14.4 ± 4.8 BCa	27.8 ± 4.0 ABa	41.1 ± 4.6 Aa	57.8 ± 3.2 Aa	34.3	<0.01
*F*	-	-	-	-	9.7	15.0	7.3	22.0	13.6	13.7		
*p*	-	-	-	-	<0.01	<0.01	<0.01	<0.01	<0.01	<0.01		

Within each row, means followed by the same uppercase letter are not significantly different (DF = 9, 89; Tukey’s HSD test at *p* = 0.05). Within each column, means followed by the same lowercase letter are not significantly different (DF = 6, 62; Tukey’s HSD test at *p* = 0.05). Where no letters exist, no significant differences were recorded. Where dashes exist, no analysis was conducted.

## Data Availability

Data are contained within the article.

## References

[1] Bakkali F., Averbeck S., Averbeck D., Idaomar M. (2008). Biological effects of essential oils—A review. Food Chem. Toxicol..

[2] Pavela R., Benelli G. (2016). Essential oils as ecofriendly biopesticides? Challenges and constraints. Trends Plant Sci..

[3] Kavallieratos N.G., Skourti A., Nika E.P., Mártonfi P., Spinozzi E., Maggi F. (2021). *Tanacetum vulgare* essential oil as grain protectant against adults and larvae of four major stored-product insect pests. J. Stored Prod. Res..

[4] Kavallieratos N.G., Nika E.P., Skourti A., Spinozzi E., Ferrati M., Petrelli R., Maggi F., Benelli G. (2022). *Carlina acaulis* essential oil: A candidate product for agrochemical industry due to its pesticidal capacity. Ind. Crops Prod..

[5] Li Y., Fabiano-Tixier A.S., Chemat F. (2014). Essential Oils: From Conventional to Green Extraction.

[6] Pavoni L., Pavela R., Cespi M., Bonacucina G., Maggi F., Zeni V., Canale A., Lucchi A., Bruschi F., Benelli G. (2019). Green micro and nanoemulsions for managing parasites, vectors and pests. Nanomaterials.

[7] Turek C., Stintzing F.C. (2013). Stability of essential oils: A review. Compr. Rev. Food Sci. Food Saf..

[8] Rees D. (2004). Insects of Stored Products.

[9] Hagstrum D.W., Klejdysz T., Subramanyam B., Nawrot J. (2013). Atlas of Stored-Product Insects and Mites.

[10] Hill D.S. (2003). Pests of Stored Foodstuffs and Their Control.

[11] Kumar R. (2017). Insect Pests on Stored Grain. Biology, Behavior, and Management Strategies.

[12] Athanassiou C.G., Kavallieratos N.G. (2014). Evaluation of spinetoram and spinosad for control of *Prostephanus truncatus, Rhyzopertha dominica, Sitophilus oryzae* and *Tribolium confusum* on stored grains under laboratory tests. J. Pest Sci..

[13] Khan T., Haider M.S., Khan H.A.A. (2022). Resistance to grain protectants and synergism in Pakistani strains of *Sitophilus oryzae* (Coleoptera: Curculionidae). Sci. Rep..

[14] Haddi K., Valbon W.R., Viteri Jumbo L.O., de Oliveira L.O., Guedes R.N.C., Oliveira E.E. (2018). Diversity and convergence of mechanisms involved in pyrethroid resistance in the stored grain weevils, *Sitophilus* spp.. Sci. Rep..

[15] Mishra B.B., Tripathi S.P., Tripathi C.P.M. (2013). Bioactivity of two plant derived essential oils against the rice weevils *Sitophilus oryzae* (L.) (Coleoptera: Curculionidae). Proc. Natl. Acad. Sci. India Sect. B.

[16] Nenaah G.E. (2014). Bioactivity of powders and essential oils of three Asteraceae plants as post-harvest grain protectants against three major coleopteran pests. J. Asia Pac. Entomol..

[17] Haouel-Hamdi S., Soltani A., Jmal R., Messaoud C., Zaouali Y., Boushih E., Jemâa J.M.B. (2021). Use of binary mixtures of three *Mentha* essential oils for the control of rice weevil *Sitophilus oryzae* (Curculionidae). Int. J. Trop. Insect Sci..

[18] Johnson A.J., Venukumar V., Varghese T.S., Viswanathan G., Leeladevi P.S., Remadevi R.K.S., Baby S. (2022). Insecticidal properties of *Clausena austroindica* leaf essential oil and its major constituent, trans-anethole, against *Sitophilus oryzae* and *Tribolium castaneum*. Ind. Crops Prod..

[19] Remesh A.V., Raveendran A., Bincy K., Wagh V.S., Dastager S.G., Babu C.S.V. (2022). Insights on biorational potential of *Ocimum gratissimum* essential oil and its binary combination with monoterpene phenol for control of rice weevil (*Sitophilus oryzae*) and aflatoxigenic fungi. Food Biosci..

[20] Yeguerman C.A., Urrutia R.I., Jesser E.N., Massiris M., Delrieux C.A., Murray A.P., Werdin González J.O. (2022). Essential oils loaded on polymeric nanoparticles: Bioefficacy against economic and medical insect pests and risk evaluation on terrestrial and aquatic non-target organisms. Environ. Sci. Pollut. Res..

[21] Zimmermann R.C., Poitevin C.G., Bischoff A.M., Beger M., da Luz T.S., Mazarotto E.J., Benatto A., Martins C.E.N., Sales Maia B.H.L.N., Sari R. (2022). Insecticidal and antifungal activities of *Melaleuca rhaphiophylla* essential oil against insects and seed-borne pathogens in stored products. Ind. Crops Prod..

[22] Saad M.M.G., El Gendy A.N.G., Elkhateeb A.M., Abdelgaleil S.A.M. (2022). Insecticidal properties and grain protective efficacy of essential oils against stored product insects. Int. J. Trop. Insect Sci..

[23] Adak T., Barik N., Patil N.B., Govindharaj G.P.P., Gadratagi B.G., Annamalai M., Mukherjee A.K., Rath P.C. (2020). Nanoemulsion of eucalyptus oil: An alternative to synthetic pesticides against two major storage insects (*Sitophilus oryzae* (L.) and *Tribolium castaneum* (Herbst)) of rice. Ind. Crops Prod..

[24] Choupanian M., Omar D., Basri M., Asib N. (2017). Preparation and characterization of neem oil nanoemulsion formulations against *Sitophilus oryzae* and *Tribolium castaneum* adults. J. Pestic. Sci..

[25] Choupanian M., Omar D. (2018). Formulation and physicochemical characterization of neem oil nanoemulsions for control of *Sitophilus oryzae* (L., 1763) (Coleoptera: Curculionidae) and *Tribolium castaneum* (Herbst, 1797) (Coleoptera: Tenebrionidae). Türk. Entomol. Derg..

[26] Tutin F.G., Heywood V.H., Burges N.A., Moore D.M., Valentine D.H., Walters S.M., Webb D.A. (1976). Flora Europaea. Plantaginaceae to Compositae (and Rubiaceae).

[27] Menale B., Amato G., Di Prisco C., Muoio R. (2006). Traditional uses of plants in North-Western Molise (Central Italy). Delpinoa.

[28] Gilca M., Tiplica G.S., Salavastru C.M. (2018). Traditional and ethnobotanical dermatology practices in Romania and other Eastern European countries. Clin. Dermatol..

[29] Abbet C., Mayor R., Roguet D., Spichiger R., Hamburger M., Potterat O. (2014). Ethnobotanical survey on wild alpine food plants in Lower and Central Valais (Switzerland). J. Ethnopharmacol..

[30] Benelli G., Pavela R., Petrelli R., Nzekoue F.K., Cappellacci L., Lupidi G., Quassinti L., Bramucci M., Sut S., Dall’Acqua S. (2019). Carlina oxide from *Carlina acaulis* root essential oil acts as a potent mosquito larvicide. Ind. Crops Prod..

[31] Benelli G., Pavoni L., Zeni V., Ricciardi R., Cosci F., Cacopardo G., Cacopardo G., Gendusa S., Spinozzi E., Petrelli R. (2020). Developing a highly stable *Carlina acaulis* essential oil nanoemulsion for managing *Lobesia botrana*. Nanomaterials.

[32] Benelli G., Rizzo R., Zeni V., Govigli A., Samková A., Sinacori M., Lo Verde G., Pavela R., Cappellacci L., Petrelli R. (2021). *Carlina acaulis* and *Trachyspermum ammi* essential oils formulated in protein baits are highly toxic and reduce aggressiveness in the medfly, *Ceratitis capitata*. Ind. Crops Prod..

[33] Pavela R., Maggi F., Petrelli R., Cappellacci L., Buccioni M., Palmieri A., Canale A., Benelli G. (2020). Outstanding insecticidal activity and sublethal effects of *Carlina acaulis* root essential oil on the housefly, *Musca domestica*, with insights on its toxicity on human cells. Food Chem. Toxicol..

[34] Pavela R., Pavoni L., Bonacucina G., Cespi M., Cappellacci L., Petrelli R., Spinozzi E., Aguzzi C., Zeppa L., Ubaldi M. (2021). Encapsulation of *Carlina acaulis* essential oil and carlina oxide to develop long-lasting mosquito larvicides: Microemulsions versus nanoemulsions. J. Pest Sci..

[35] Spinozzi E., Ferrati M., Cappellacci L., Caselli A., Perinelli D.R., Bonacucina G., Maggi F., Strzemski M., Petrelli R., Pavela R. (2023). *Carlina acaulis* L. (Asteraceae): Biology, phytochemistry, and application as a promising source of effective green insecticides and acaricides. Ind. Crops Prod..

[36] Lange B.M., Croteau R. (1999). Genetic engineering of essential oil production in mint. Curr. Opin. Plant Biol..

[37] Sevindik M., Akgul H., Pehlivan M., Selamoglu Z. (2017). Determination of therapeutic potential of *Mentha longifolia* ssp. longifolia. Fresenius Environ. Bull..

[38] Jamzad Z. (2012). Flora of Iran: Lamiaceae.

[39] Durrani F., Sultan A., Marri M.L., Chand N., Durrani Z. (2007). Effect of wild mint (*Mentha longifolia*) infusion on the over all performance of broiler chicks. Pak. J. Biol. Sci..

[40] Al-Bayati F.A. (2009). Isolation and Identification of Antimicrobial Compound from *Mentha Longifolia* L. Leaves Grown Wild in Iraq. Ann. Clin. Microbiol. Antimicrob..

[41] Mkaddem M., Bouajila J., Ennajar M., Lebrihi A., Mathieu F., Romdhane M. (2009). Chemical composition and antimicrobial and antioxidant activities of *Mentha* (*longifolia* L. and *viridis*) essential oils. J. Food Sci..

[42] Bahadori M.B., Zengin G., Bahadori S., Dinparast L., Movahhedin N. (2018). Phenolic composition and functional properties of wild mint (*Mentha longifolia* var. *calliantha* (Stapf) Briq.). Int. J. Food Prop..

[43] Gharib F.A., Mansour K.H., Ahmed E.Z., Galal T.M. (2021). Heavy metals concentration, and antioxidant activity of the essential oil of the wild mint (*Mentha longifolia* L.) in the Egyptian watercourses. Int. J. Phytoremediation.

[44] Khani A., Asghari J. (2012). Insecticide activity of essential oils of *Mentha longifolia, Pulicaria gnaphalodes* and *Achillea wilhelmsii* against two stored product pests, the flour beetle, *Tribolium castaneum*, and the cowpea weevil, *Callosobruchus maculatus*. J. Insect Sci..

[45] Farzaei M.H., Bahramsoltani R., Ghobadi A., Farzaei F., Najafi F. (2017). Pharmacological activity of *Mentha longifolia* and its phytoconstituents. J. Trad. Chin. Med..

[46] Benelli G., Pavela R., Rakotosaona R., Nzekoue F.K., Canale A., Nicoletti M., Maggi F. (2020). Insecticidal and mosquito repellent efficacy of the essential oils from stem bark and wood of *Hazomalania voyronii*. J. Ethnopharmacol..

[47] Benelli G., Maggi F., Petrelli R., Canale A., Nicoletti M., Rakotosaona R., Rasoanaivo P. (2017). Not ordinary antimalarial drugs: Madagascar plant decoctions potentiating the chloroquine action against *Plasmodium* parasites. Ind. Crops Prod..

[48] Ratsimamanga-Urverg S., Rasoanaivo P., Milijaona R., Rakotoarimanga J., Rafatro H., Robijaona B., Rakoto-Ratsimamanga A., Verdier F., Le Bras J. (1994). In vitro antimalarial activity, chloroquine potentiating effect and cytotoxicity of alkaloids of *Hernandia voyronii* Jum.(Hernandiaceae). Phytother. Res..

[49] Kavallieratos N.G., Nika E.P., Skourti A., Ntalli N., Boukouvala M.C., Ntalaka C.T., Maggi F., Rakotosaona R., Cespi M., Perinelli D.R. (2021). Developing a *Hazomalania voyronii* essential oil nanoemulsion for the eco-friendly management of *Tribolium confusum, Tribolium castaneum* and *Tenebrio molitor* larvae and adults on stored wheat. Molecules.

[50] Kavallieratos N.G., Nika E.P., Skourti A., Boukouvala M.C., Ntalaka C.T., Maggi F., Spinozzi E., Petrelli R., Perinelli D.R., Benelli G. (2022). *Carlina acaulis* essential oil nanoemulsion as a new grain protectant against different developmental stages of three stored-product beetles. Pest Manag. Sci..

[51] Kavallieratos N.G., Nika E.P., Skourti A., Xefteri D.N., Cianfaglione K., Perinelli R., Spinozzi E., Bonacucina G., Canale A., Benelli G. (2022). Piperitenone oxide-rich *Mentha longifolia* essential oil and its nanoemulsion to manage different developmental stages of insect and mite pests attacking stored wheat. Ind. Crops Prod..

[52] Kavallieratos N.G., Boukouvala M.C., Ntalli N., Skourti A., Karagianni E.S., Nika E.P., Kontodimas D.C., Cappellacci L., Petrelli R., Cianfaglione K. (2020). Effectiveness of eight essential oils against two key stored-product beetles, *Prostephanus truncatus* (Horn) and *Trogoderma granarium* Everts. Food Chem. Toxicol..

[53] Attia M.A., Wahba T.F., Shaarawy N., Moustafa F.I., Guedes R.N.C., Dewer Y. (2020). Stored grain pest prevalence and insecticide resistance in Egyptian populations of the red flour beetle *Tribolium castaneum* (Herbst) and the rice weevil *Sitophilus oryzae* (L.). J. Stored Prod. Res..

[54] Zakladnoy G.A. (2020). The first detection of resistance to phosphine in a natural population of the rice weevil *Sitophilus oryzae* (L.) (Coleoptera, Dryophthoridae) in Russia. Entomol. Rev..

[55] Khan T., Khan H.A.A., Haider M.S., Anwar W., Akhter A. (2022). Selection for resistance to pirimiphos-methyl, permethrin and spinosad in a field strain of *Sitophilus oryzae*: Resistance risk assessment, cross-resistance potential and synergism of insecticides. Environ. Sci. Pollut. Res..

[56] Mehta V., Surjeet K. (2020). Influence of different plant powders as grain protectants on *Sitophilus oryzae* (L.) (Coleoptera: Curculionidae) in stored wheat. J. Food Prot..

[57] Chaudhuri N., Subba B. (2008). Evaluation of efficacy of different grain protectants against *Sitophilus oryzae* infesting paddy, wheat and maize grains. Int. J. Curr. Microbiol. App. Sci..

[58] Yankanchi S.R., Gadache A.H. (2010). Grain protectant efficacy of certain plant extracts against rice weevil, *Sitophilus oryzae* L. (Coleoptera: Curculionidae). J. Biopestic..

[59] Minto R.E., Blacklock B.J. (2008). Biosynthesis and function of polyacetylenes and allied natural products. Prog. Lipid Res..

[60] Arnason T., Swain T., Wat C.K., Graham E.A., Partington S., Towers G.H.N., Lam J. (1981). Mosquito larvicidal activity of polyacetylenes from species in the Asteraceae. Biochem. Syst. Ecol..

[61] Waksmundzka-Hajnos M., Sherma J., Kowalska T. (2008). Thin Layer Chromatography in Phytochemistry.

[62] Wink M. (2012). Medicinal plants: A source of anti-parasitic secondary metabolites. Molecules.

[63] Božović M., Pirolli A., Ragno R. (2015). *Mentha suaveolens* Ehrh. (Lamiaceae) essential oil and its main constituent piperitenone oxide: Biological activities and chemistry. Molecules.

[64] Tripathi A.K., Prajapati V., Ahmad A., Aggarwal K.K., Khanuja S.P. (2004). Piperitenone oxide as toxic, repellent, and reproduction retardant toward malarial vector *Anopheles stephensi* (Diptera: Anophelinae). J. Med. Entomol..

[65] Wink M., Schimmer O. (1999). Modes of action of defensive secondary metabolites. Annu. Plant Rev..

[66] Oboh G., Ademosun A.O., Olumuyiwa T.A., Olasehinde T.A., Ademiluyi A.O., Adeyemo A.C. (2017). Insecticidal activity of essential oil from orange peels (*Citrus sinensis*) against *Tribolium confusum, Callosobruchus maculatus* and *Sitophilus oryzae* and its inhibitory effects on acetylcholinesterase and Na+/K+-ATPase activities. Phytoparasitica.

[67] Coats J., Karr L., Drewes C., Hedin P.A. (1991). Toxicity and neurotoxic effects of monoterpenoids: In insects and earthworms. Naturally Occurring Pest Bioregulators.

[68] Park C.G., Jang M., Yoon K.A., Kim J. (2016). Insecticidal and acetylcholinesterase inhibitory activities of Lamiaceae plant essential oils and their major components against *Drosophila suzukii* (Diptera: Drosophilidae). Ind. Crops Prod..

[69] Lee S.E., Lee B.H., Choi W.S., Park B.S., Kim J.G., Campbell B.C. (2001). Fumigant toxicity of volatile natural products from Korean spices and medicinal plants towards the rice weevil, *Sitophilus oryzae* (L). Pest Manag. Sci..

[70] Lee B.H., Annis P.C., Choi W.S. (2004). Fumigant toxicity of essential oils from the Myrtaceae family and 1, 8-cineole against 3 major stored-grain insects. J. Stored Prod. Res..

[71] Ryan M.F., Byrne O. (1988). Plant-insect coevolution and inhibition of acetylcholinesterase. J. Chem. Ecol..

[72] Kavallieratos N.G., Michail E.J., Boukouvala M.C., Nika E.P., Skourti A. (2019). Efficacy of pirimiphos-methyl, deltamethrin, spinosad and silicoSec against adults and larvae of *Tenebrio molitor* L. on wheat, barley and maize. J. Stored Prod. Res..

[73] Kavallieratos N.G., Athanassiou C.G., Diamantis G.C., Gioukari H.G., Boukouvala M.C. (2017). Evaluation of six insecticides against adults and larvae of *Trogoderma granarium* Everts (Coleoptera: Dermestidae) on wheat, barley, maize and rough rice. J. Stored Prod. Res..

[74] Arthur F.H. (2016). Efficacy of methoprene for multi-year protection of stored wheat, brown rice, rough rice and corn. J. Stored Prod. Res..

[75] Boukouvala M.C., Kavallieratos N.G., Nika E.P. (2022). Insecticidal properties of etofenprox for the control of *Ephestia kuehniella, Rhyzopertha dominica, Sitophilus oryzae,* and *Tribolium confusum* on stored barley, maize, oats, rice, and wheat. Environ. Sci. Pollut. Res..

[76] Pires E.M., Nogueira R.M., Pina D.S., Manica C.L.M., Faroni L.R.A., Moreira P.S.A. (2016). Walking stability of *Rhyzopertha dominica* (Fabricius, 1792) (Coleoptera: Bostrichidae). Braz. J. Biol..

[77] Boukouvala M.C., Kavallieratos N.G., Athanassiou C.G., Losic D., Hadjiarapoglou L.P., Elemes Y. (2017). Laboratory evaluation of five novel pyrrole derivatives as grain protectants against *Tribolium confusum* and *Ephestia kuehniella* larvae. J. Pest Sci..

[78] Losic D., Korunic Z., Losic D. (2018). Diatomaceous earth, a natural insecticide for stored grain protection: Recent progress and perspectives. Diatom Nano-technology: Progress and Emerging Applications.

[79] Kavallieratos N.G., Athanassiou C.G., Pashalidou F.G., Andris N.S., Tomanović Ž. (2005). Influence of grain type on the insecticidal efficacy of two diatomaceous earth formulations against *Rhyzopertha dominica* (F) (Coleoptera: Bostrychidae). Pest Manag. Sci..

[80] Kavallieratos N.G., Athanassiou C.G., Nika E.P., Boukouvala M.C. (2017). Efficacy of alpha-cypermethrin, chlorfenapyr and pirimiphos-methyl applied on polypropylene bags for the control of *Prostephanus truncatus* (Horn), *Rhyzopertha dominica* (F.) and *Sitophilus oryzae* (L.). J. Stored Prod. Res..

[81] Kar S., Nayak R.N., Sahoo N.R., Bakhara C.K., Panda M.K., Pal U.S., Bal L.M. (2021). Rice weevil management through application of silica nano particle and physicochemical and cooking characterization of the treated rice. J. Stored Prod. Res..

[82] Zar J.H. (2014). Biostatistical Analysis.

[83] Scheff D.S., Arthur F.H. (2018). Fecundity of *Tribolium castaneum* and *Tribolium confusum* adults after exposure to deltamethrin packaging. J. Pest Sci..

[84] Sall J., Lehman A., Creighton L. (2001). JMP start statistics. A Guide to Statistics and Data Analysis Using JMP and JMP in Software.

[85] SAS Institute Inc. (2021). Using JMP 16.2.

[86] Sokal R.R., Rohlf F.J. (1995). Biometry.

[87] Arthur F.H. (1996). Grain protectants: Current status and prospects for the future. J. Stored Prod. Res..

[88] Benelli G., Ceccarelli C., Zeni V., Rizzo R., Lo Verde G., Sinacori M., Boukouvala M.C., Kavallieratos N.G., Ubaldi M., Tomassoni D. (2022). Lethal and behavioural effects of a green insecticide against an invasive polyphagous fruit fly pest and its safety to mammals. Chemosphere.

